# The Immunolocalization of Mast Cells in the Pathology of Oral Submucous Fibrosis

**DOI:** 10.7759/cureus.40069

**Published:** 2023-06-06

**Authors:** Seema Gupta, Manish Sharma, Satyabrat Banerjee, Kiran Holikatti, Priyanka Kamble, Jay V Goyal

**Affiliations:** 1 Orthodontics, Jawahar Medical Foundation’s (JMF) Annasaheb Chudaman Patil Memorial (ACPM) Dental College, Dhule, IND; 2 Oral Pathology and Microbiology, Jawahar Medical Foundation’s (JMF) Annasaheb Chudaman Patil Memorial (ACPM) Dental College, Dhule, IND; 3 Conservative Dentistry and Endodontics, Jawahar Medical Foundation’s (JMF) Annasaheb Chudaman Patil Memorial (ACPM) Dental College, Dhule, IND; 4 Oral and Maxillofacial Surgery, Jawahar Medical Foundation’s (JMF) Annasaheb Chudaman Patil Memorial (ACPM) Dental College, Dhule, IND

**Keywords:** cd117 expression, atrophy, mast cells, vascularity, premalignancy, oral submucous fibrosis

## Abstract

Background

Oral submucous fibrosis (OSMF) is a prevalent precancerous condition of the oral cavity and an ambiguity to clinicians because of its indistinguishable etiopathogenesis. Previous studies could not establish a definite role of mast cells (MCs) in the fibrosis of stroma. The present study was done to study the histopathological changes in OSMF and to determine the association of mast cells (MCs) and their degranulated components with vascularity.

Methods

A retrospective case-control study involved 40 cases of various histopathological grades of OSMF and was compared with 10 cases of normal buccal mucosa by using a cluster of differentiation 117* *(CD117) kit for the identification of MCs and Masson’s trichrome stain to study the number of blood vessels (BVs).

Results

The present study indicated that advanced cases of OSMF had keratinized epithelium with atrophic changes and moderate to advanced fibrosis of stroma with the involvement of underlying muscles. The MC density and the number of blood vessels were progressively reduced in OSMF as the grade advanced compared to healthy controls.

Conclusion

An increase in the mast cell density in the initial stages of OSMF suggests their definite role in the initiation of fibrosis and secondary changes to the epithelium such as atrophy.

## Introduction

Oral submucosal fibrosis (OSMF) is a progressive, chronic inflammatory condition that affects the oral mucosa and surrounding structures. It is a predominant oral potentially malignant disorder caused by the betel quid habit [1]. It is considered ambiguous to clinicians because many aspects of its etiopathogenesis are still incomprehensible, thereby accounting for extensive research in this field. Various etiological factors are considered in the causation of OSMF, such as chilies, malnutrition, autoimmune etiology, and areca nut alkaloids [2]. The early stages of OSMF are characterized by the presence of signs and symptoms of a burning sensation in the mouth, ulcerations, and recurrent stomatitis. The later stages include stiffness of the oral mucosa, trismus, and reduced mobility of the tongue and soft palate due to progressive fibrosis. In extreme cases, the patients may experience progressive dysphagia [2].

Mast cells (MCs) are indistinguishable components of connective tissue and other inflammatory cells and are associated with several inflammatory disorders, including lichen planus, gingivitis, pulpitis, and aggressive periodontitis [3,4]. MCs are known to cause a burning sensation in OSMF, and more mast cells are detected in the early stages of OSMF than in the advanced stages of OSMF [5]. MCs have been implicated in fibrosis of the stroma, leading to malignant changes in the OSMF [6]. The degree of vascularity also decreases as the disease progresses [7]. By understanding the role of various factors such as MCs in the etiology of OSMF, we can convert our knowledge of the etiology of OSMF into treatment approaches.

Various methods, such as toluidine blue and alcian blue, stain mast cells by metachromatic staining. The incompetency of toluidine blue is the impartial staining of basophils in tissue sections [8]. The receptor for promoting mast cell development and differentiation, also known as mast cell growth factor, is a type III tyrosine kinase protein called cluster of differentiation 117 (CD117) that is expressed by melanocytes, germ cells, and mast cell progenitors. This marker has been consistently used for mast cell localization in various mast cell-related disorders and neoplastic conditions [9-11]. Owing to the limited literature available, this study was conducted to determine the association between mast cells and vascularity in different histological grades of OSMF. The objectives of this study were to determine the mean density of MCs and blood vessels (BVs) in the OSMF and control groups, to study different stages of OSMF histologically, and to find any association between MCs and vascularity in different grades of OSMF.

## Materials and methods

A case-control retrospective study was conducted on 40 paraffin-embedded tissue sections, histologically diagnosed using hematoxylin and eosin (H&E) staining as cases of oral submucous fibrosis, selected from the department of oral pathology. Using Pindborg’s criteria, OSMF cases were histopathologically graded into four grades: very early to advanced [4]. Forty cases (10 per grade) were selected for this study. For comparison, 10 tissue samples of the normal healthy oral mucosa (buccal mucosa) procured from age- and sex-matched subjects with no habits constituted the control group. The control group participants explained the study in detail, and the patients consented to participate in the study and signed an informed consent form before the commencement of the study. The study was approved by the Institutional Ethical Committee (IEC) of Jawahar Medical Foundation’s (JMF) Annasaheb Chudaman Patil Memorial (ACPM) Dental College with the reference number ECR/1448/inst/MH/2022/021 and followed the principles of the Declaration of Helsinki.

Histological examination

**Figure 1:**
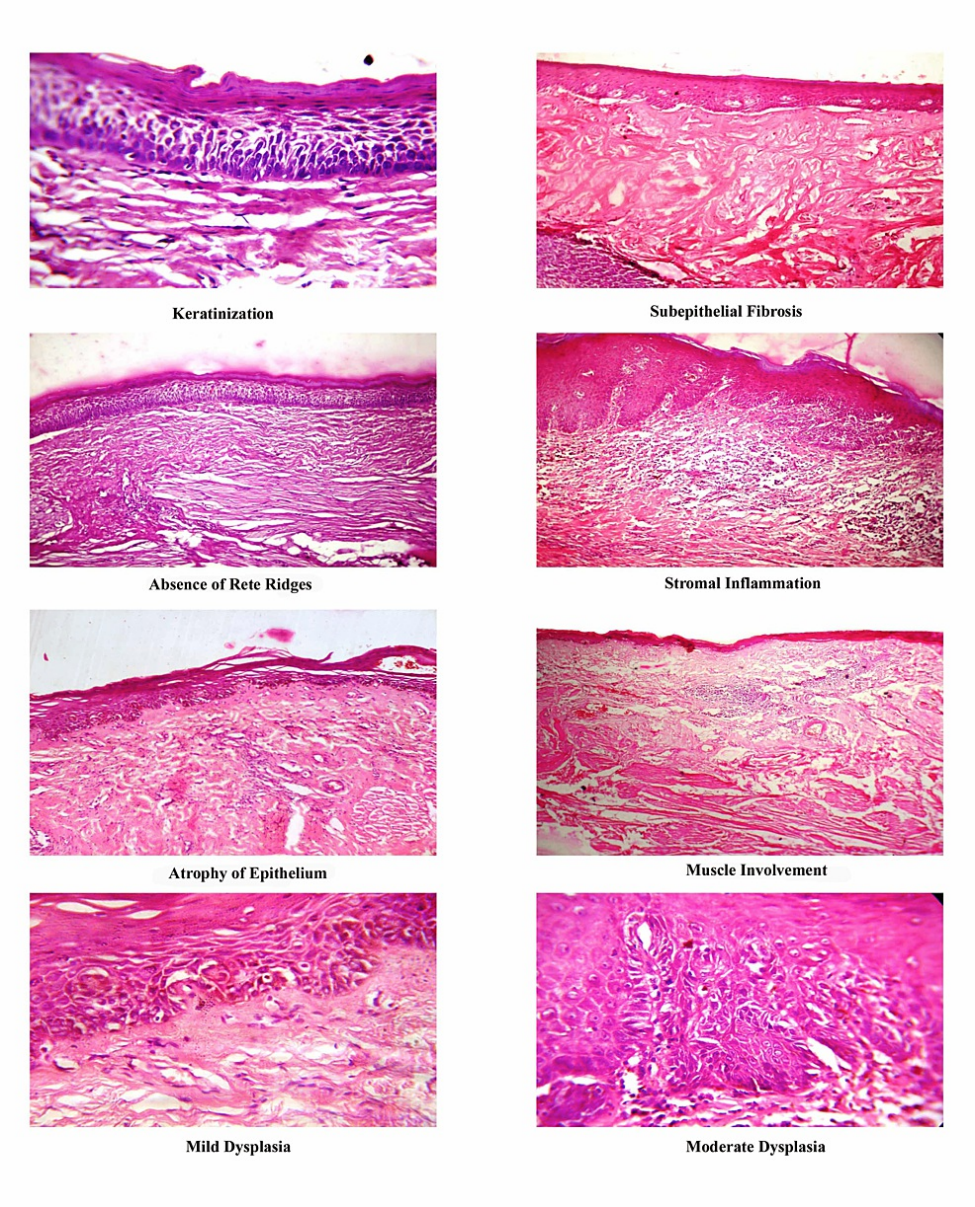
Histopathological features of different grades of OSMF in hematoxylin and eosin-stained sections OSMF: oral submucous fibrosis

**Figure 2:**
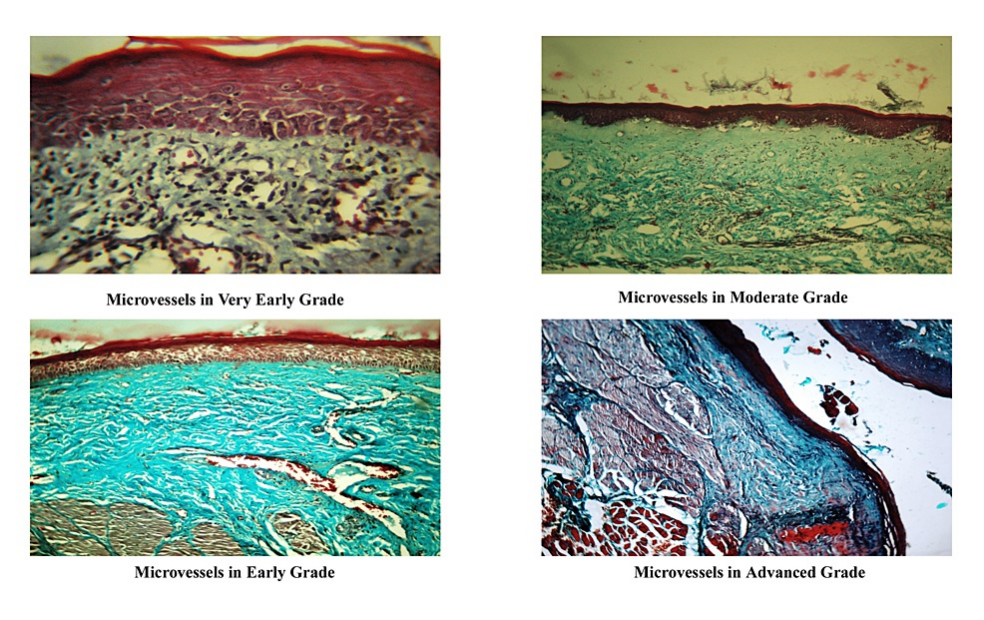
Assessment of microvessels in different grades of OSMF in Masson’s trichrome stain OSMF: oral submucous fibrosis

The H&E sections were studied in detail by two independent oral pathologists for the changes in the epithelium and connective tissue. The epithelium was examined for the presence or absence of keratinization, atrophy, rete ridges, and dysplasia, and the connective tissue was assessed for inflammation, stromal fibrosis, and underlying muscle involvement (Figure 1). Masson’s trichrome-stained sections were analyzed for the density of microvessels in the stroma (Figure 2).

Immunohistochemical staining of MCs

**Figure 3:**
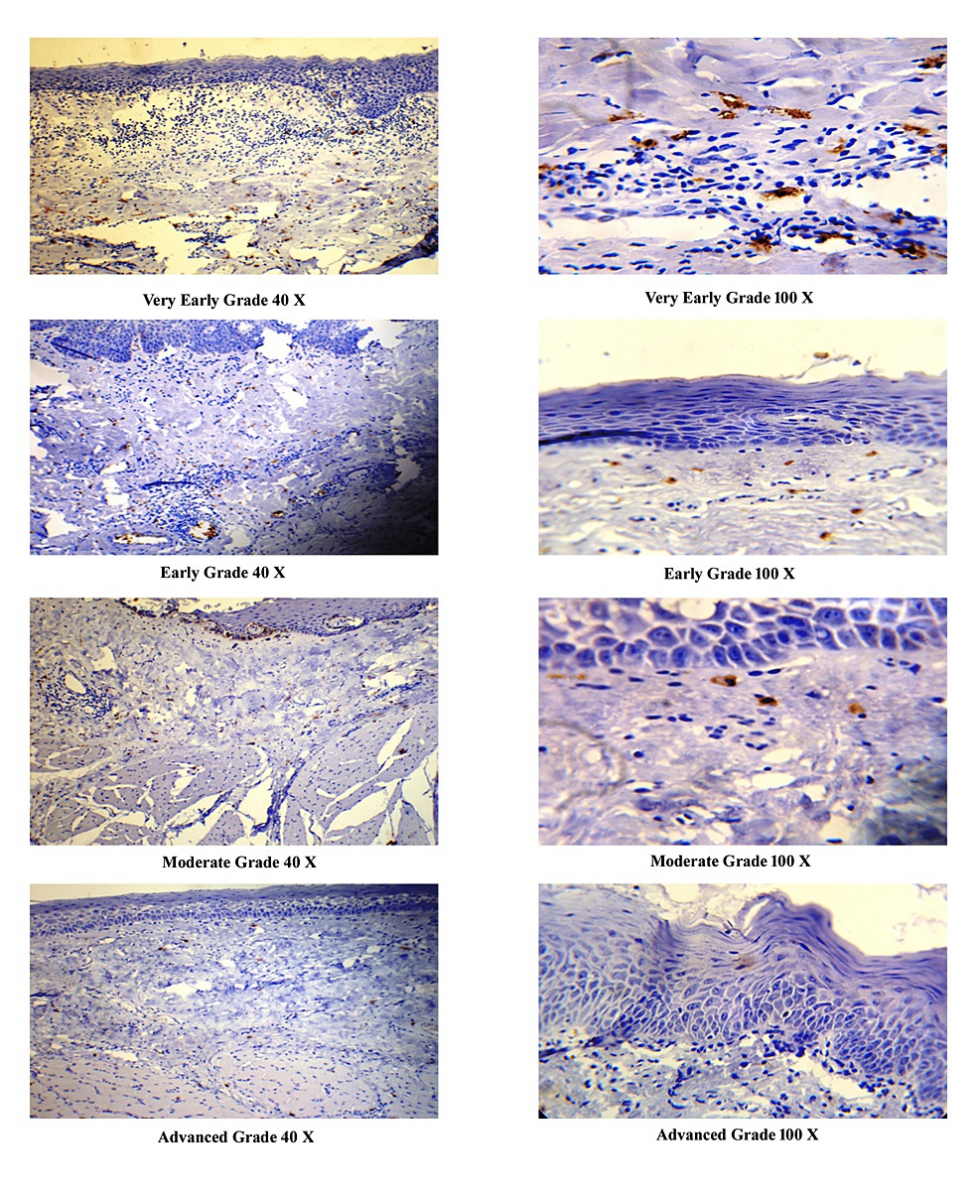
Immunolocalization of mast cells with CD117 marker in different grades of OSMF at 40× and 100× magnification CD117, cluster of differentiation 117; OSMF, oral submucous fibrosis

For the evaluation of MCs, 4 µm-thick sections were taken on poly-L-lysine (PLL) slides and stained with polyclonal rabbit antihuman CD117 (c-kit 1:400, Dako, Carpinteria, CA) using a standard avidin-biotin-peroxidase complex method (Figure 3).

Assessment of mast cell and vascular density

Two oral pathologists independently examined the stained sections. The stroma was divided into two zones: the subepithelial zone and the deep zone. Each zone was considered separately, and the number of mast cells and blood vessels was counted using 40× microscope objectives. Five random nonoverlapping fields were selected by two oral pathologists who were blinded to the diagnosis of the cases. The average score of the five fields was recorded for data analysis.

Statistical analysis

The data were collected, transferred to Microsoft Excel (Microsoft® Corp., Redmond, WA), and sent for statistical analysis. The obtained data passed the Shapiro-Wilk test for normal distribution; therefore, parametric tests were used for analysis. Inter-observer reliability was evaluated by inter-rater agreement in the histopathological assessment of OSMF. Analysis of variance (ANOVA) was used to compare the mean number of mast cells and blood vessels in the different grades of OSMF and control groups. A p-value of less than or equal to 0.05 was considered statistically significant.

## Results

Demographic characteristics

The study included 50 samples: 40 OSMF (80%) cases and 10 (20%) healthy controls. Of the 40 cases, 10 of each histopathological grade of OSMF were considered. The average age was 32.8±8.5 years (minimum of 16 and maximum of 49 years) for OSMF cases and 29.4±9.2 years (minimum of 17 and maximum of 47 years) for controls. The study noted a marked sex difference with overall male dominance in all age groups, as the male-to-female ratio was 4:1.

Inter-rater reliability

The quantification of mast cells and blood vessels (BVs) and histological examination were performed by two independent observers. The inter-rater reliability was 98% for MCs and 92% for BVs, which shows excellent agreement (Table 1).

**Table 1:** Inter-observer reliability for mast cell and blood vessel quantification in study samples *p≤0.05, significant; **p≤0.001, highly significant; ***p≤0.0001, very highly significant

Inter-observer reliability				
		Data pairs	r-value	p-value
Mast cells	Observer 1	50	0.98	0.0001***
	Observer 2			
Blood vessels	Observer 1	50	0.92	0.0001***
	Observer 2			

**Figure 4:**
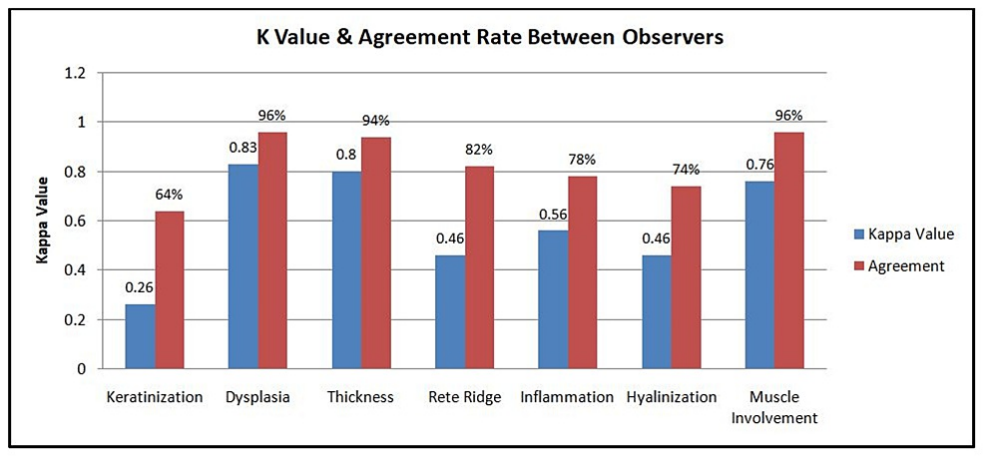
Graph showing kappa agreement between two observers for different histological parameters

For histopathological examination, most of the parameters showed fair to the perfect agreement (kappa value ranged from 0.26 to 0.76) (Figure 4).

Distribution of mast cells and blood vessels in tissue zones

**Figure 5:**
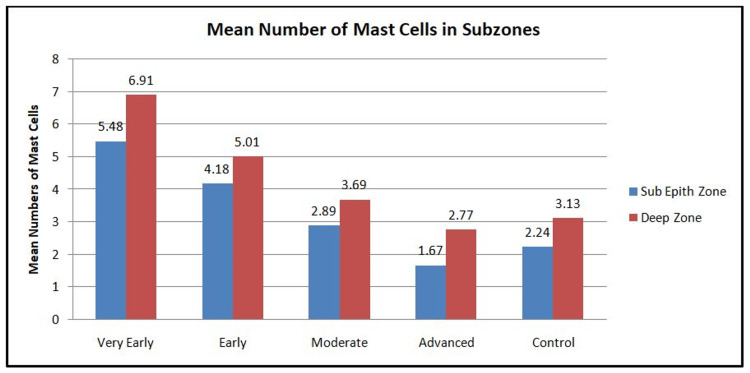
Graph showing the mean number of mast cells in different grades of OSMF and control in subepithelial and deep zones OSMF: oral submucous fibrosis

**Figure 6:**
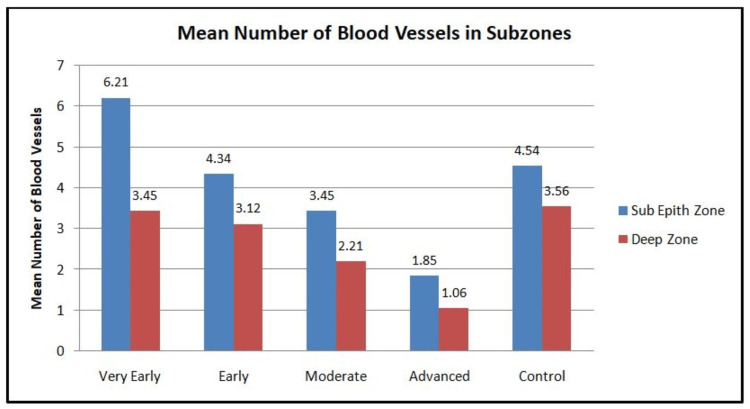
Graph showing the mean number of blood vessels in different grades of OSMF and control in subepithelial and deep zones OSMF: oral submucous fibrosis

The distribution of mast cells and blood vessels was studied in subepithelial and deeper zones of tissue sections (Figures 5-6).

The cumulative number of CD117-stained mast cells in the subepithelial zone was less than that in the deeper zone for all grades of OSMF and in the control group, whereas for blood vessels, it was more in the subepithelial zone than in the deep zone. On the evaluation of different grades of OSMF, in both zones, there was a statistically significant difference in the number of MCs and BVs as compared to the control (p<0.0001), with a maximum number of mast cells and blood vessels seen in very early grade and the least in advanced grade (Table 2).

**Table 2:** ANOVA test for mast cell and blood vessel number between different grades of OSMF and control in tissue zones *p≤0.05, significant; **p≤0.001, highly significant; ***p≤0.0001, very highly significant OSMF, oral submucous fibrosis; ANOVA, analysis of variance

ANOVA test							
Histological grades of OSMF	Sample size (N)	Mean (SD) mast cells			Mean (SD) blood vessels		
		Subepithelial	Deep	p-value	Subepithelial	Deep	p-value
Very early	10	5.475±0.60	6.90±0.17	0.001**	6.21±0.45	3.45±0.19	0.001**
Early	10	4.18±0.38	5.01±0.33		4.34±0.34	3.12±0.38	
Moderate	10	2.89±0.17	3.69±0.23		3.45±0.23	2.21±0.36	
Advanced	10	1.67±0.31	2.765±0.20		1.85±0.28	1.06±0.42	
Control	10	2.24±0.28	3.13±0.31		4.54±0.38	3.56±0.21	

Histopathological features in different grades of OSMF and control

The advanced stages of OSMF had keratinized atrophic epithelium, absent rete ridges with dysplastic changes, and fibrosis with muscle involvement in the stroma, while early stages showed signs of inflammation, compared to the control (Table 3).

**Table 3:** Cohen kappa (K) reliability for histopathological features in different grades of OSMF and control OSMF: oral submucous fibrosis

Histological grades of OSMF	Sample size (N)	Keratinization	Dysplasia	Rete ridge	Atrophy	Inflammation	Fibrosis	Muscle involvement
Very early	10	3 (30%)	0 (0%)	10 (100%)	0 (0%)	10 (100%)	1 (10%)	0 (0%)
Early	10	5 (50%)	1 (10%)	10 (100%)	0 (0%)	8 (80%)	4 (40%)	0 (0%)
Moderate	10	5 (50%)	2 (20%)	8 (80%)	2 (20%)	5 (50%)	6 (60%)	4 (40%)
Advanced	10	8 (80%)	3 (30%)	4 (40%)	6 (60%)	2 (20%)	9 (90%)	7 (70%)
Control	10	2 (20%)	0 (0%)	10 (100%)	0 (0%)	2 (20%)	0 (0%)	0 (0%)
K-value		0.26	0.83	0.8	0.46	0.56	0.46	0.76

Association between MCs and BVs in different grades of OSMF and control

The unpaired T test revealed a nonsignificant association between MC density and BV density in OSMF and control (p≥0.05) (Table 4).

**Table 4:** Unpaired T test between mast cell and blood vessel numbers between different grades of OSMF and control OSMF: oral submucous fibrosis

Histological grades of OSMF	Mast cell mean	Blood vessel mean	p-value
Very early	12.38±0.72	9.66±0.60	0.663
Early	9.19±0.48	7.46±0.34	
Moderate	6.58±0.27	5.66±0.47	
Advanced	4.435±0.43	2.91±0.28	
Control	5.37±0.53	8.1±0.56	

## Discussion

OSMF is a widespread precancerous condition that affects the oral cavity and leads to blanching of mucosal tissues, burning sensation of the mucosa, stromal fibrosis, and restricted mouth opening [1,2].

Histopathological features of OSMF

In the histological tissue sections of the OSMF, keratinization and atrophy of the epithelium with the loss of rete ridges were predominant in advanced stages. The basic etiology of epithelial atrophy has been suggested by many researchers, but it is still considered an assumption and a hypothesis [12]. Epithelial atrophy in the progressive stages of OSMF leads to the loss of barrier protection, which may be due to morphological changes in the epithelial cells secondary to stromal effects [13]. Early grades of OSMF are characterized by the presence of inflammation in the stroma, which progresses to fibrosis and muscle involvement in later grades. On histopathological evaluation, it is reasonable that stromal changes may play a role in inducing the epithelial changes seen in OSMF and might therefore be involved in the malignant changes that occur in OSMF cases [14]. Epithelial impairment may be due to connective tissue changes such as fibrosis, reduced vascularity, and muscle changes, which were found to be present in 70%-90% of advanced cases of OSMF, which was in accordance with previous studies [12]. Muscle changes might be due to a degenerative process involving muscle fibers and surrounding connective tissue. This was characterized by scanty collagen fibrils and the accumulation of edematous-like fluid around these muscles. In our study, we chose Masson’s trichrome stain, as this offers a simultaneous contrast color to the collagen fibers from the muscles. Collagen was stained green, while the muscle had a brilliant red color. This color contrast facilitates better visual discrimination between muscle and collagen.

Angiogenesis, mast cells, and OSMF

In routine H&E-stained histological sections of OSMF, it is not feasible to locate mast cells as different cells from basophils. The same problem is often encountered with toluidine blue, which stains basophils with the same intensity in tissue sections. Previous studies using toluidine blue to quantify mast cells at different stages of OSMF have shown results with arguable differences [9]. These perceptible differences may be due to other granular inflammatory cells that had been stained with toluidine blue and overdiagnosed as mast cells. Another reason may be that the mast cells in these cases may have been degranulated as the disease progressively advances. Toluidine blue stains mast cell granules; therefore, the lack of granules in mast cells of advanced grades may not have been stained with toluidine blue. As reported by Khatri et al. [9], there were no significant differences in MCs, compared to the control in advanced grades of OSMF when stained with toluidine blue, but with c-kit, significant differences were noted with decreasing number of MCs with advanced stages of OSMF. Therefore, to minimize this discrepancy, we employed a specific marker (CD117) for the categorical separation of mast cells from other cells and to stain degranulated mast cells in our study. CD117-stained mast cells were seen in subepithelial and deeper zone of connective tissue in the very early, early, moderate, and advanced grades of OSMF at 40× and 100× magnification (Figure 3). The majority of cells were found in the vicinity of blood vessels as suggested by Tekade et al. [7].

In our study, we found a significant increase in MCs and vascular density in the early grades of OSMF and a gradual decrease as the grade progressed. Continuous chronic irritation due to chewing of areca nuts may cause MC activation and degranulation, releasing inflammatory cell mediators such as histamine, tryptase, heparin, histamine, serotonin acid hydrolases, and cytokines such as tumor necrosis factor-alpha (TNF-α) and interleukin 16 (IL-16), causing the initial inflammatory signs of burning sensation, stomatitis, and glossitis in OSMF [15]. Several authors have proposed that mast cell mediators also contribute to the vasodilation effect and increase vascularity in the initial grades of OSMF [7].

In the present study, Masson trichrome stain has been used to study blood vessels as it effectively stains collagen and smooth muscle fibers, which are present in the walls of blood vessels and, hence, useful in studying vessel wall changes [16]. The predominant presence of dilated and congested vessels in the OSMF cases in our study (65%) indicated that vascular dilatation could be due to inflammatory mediators, such as histamine, released by the degranulation of MCs. The degree of vascularity of the diseased mucosa and its effect on epithelial thickness in OSMF are controversial and have always been a matter of debate with conflicting results [17,18].

Mast cell-mediated tissue fibrosis is due to the releases of TGF-β that activates monocytes involved in angiogenesis and fibrosis. In addition, activated mast cells release chemical mediators, TGF and angiotensin II, which are also involved in fibrotic processes. The increase in vascularity in the early grades of OSMF could be due to inflammatory mediator [7]. The reason behind the significant decrease in MCs and vascularity in the later grades of OSMF can be attributed to progressive fibrosis in the stroma, which reduces the vascularity and production of MCs. The role of MCs in fibrosis is controversial, and MCs are known to release mediators that promote fibrogenesis [6,18,19]. Our findings are consistent with previous studies [3,5,17]. Our findings disagree with those of Rajendran et al., who observed similar values of the mean vascular density in various grades of OSMF, which were marginally lower than those of normal oral mucosa [20]. Pujari and Vidya found an increase in mean MC density even in advanced grades of OSMF [21]. This disparity might be due to differences in sample size, dye used to study vascular density, and markers used to study MCs.

Limitations of the study

The present study used Masson’s trichrome staining to assess the number of blood vessels; therefore, studies assessing vascularity using immunohistochemical markers are more reliable and informative. Further studies with a larger sample size are recommended to assess the definitive role of MCs in OSMF.

Clinical implications of the study

Based on the findings of the current study, strategies may be directed toward MCs to control the progression of OSMF. Based on the concept that MCs play a role in chronic inflammation, releasing mediators to cause vasodilation, and probable fibrosis of the stroma, the use of MC stabilizers can be effective in controlling the progression of OSMF. However, more research is needed to assess the role of mast cells in fibrosis in OSMF. Detailed histopathological examination also helps in recognizing early changes in patients with OSMF, thereby promoting early interventional therapy to prevent progression to malignant oral squamous cell carcinoma.

## Conclusions

The present study supports previous findings of the literature that vascularity and MC density increases progressively from normal mucosa to OSMF in the initial stages, whereas in the progressive stages of OSMF, there was a reduction in vascularity and number of MCs, which may cause atrophic changes in the overlying epithelium and subsequent malignant transformation.
